# Optimized ultrasound extraction and cytotoxic activity against HeLa cervical cancer cells of total flavonoids of *Physochlaina physaloides* (L.) G. Don

**DOI:** 10.3389/fphar.2026.1800105

**Published:** 2026-04-08

**Authors:** Zhi Ying Sun, Jie Xu, Wei Wei

**Affiliations:** 1 College of Life Sciences and Technology, Inner Mongolia Normal University, Hohhot, Inner Mongolia, China; 2 Key Laboratory of Biodiversity Conservation and Sustainable Utilization in Mongolian Plateau, Colleges and Universities of Inner Mongolia Autonomous Region, Hohhot, China; 3 State Key Laboratory of Chinese Medicine Modernization, Tianjin University of Traditional Chinese Medicine, Tianjin, China

**Keywords:** cytotoxicity, flavonoids, *Physochlaina physaloides* (L.) G. Don, response surface methodology, ultrasound-assisted extraction

## Abstract

**Introduction:**

*Physochlaina physaloides* (L.) G. Don is a traditionally used botanical drug whose antitumor activity has been linked to flavonoid-enriched fractions. This study aimed to optimize the ultrasound-assisted extraction (UAE) of total flavonoids from the roots of P. physaloides and to evaluate the cytotoxic activity of the resulting optimized flavonoid extract (OFE) in human cervical cancer cells.

**Methods:**

Total flavonoids were prepared from the roots of *P. physaloides* using UAE. A Box–Behnken response-surface design was employed to optimize the extraction conditions. The effects of the solid-liquid ratio, extraction temperature, and ultrasonic power on flavonoid yield were assessed. The bioactivity of OFE was then evaluated in HeLa cells using the MTT assay, Calcein-AM/PI staining, Annexin V-FITC/PI apoptosis analysis, and western blotting of apoptosis- and survival-associated proteins.

**Results:**

The solid–liquid ratio, temperature, and ultrasonic power were identified as the key factors influencing flavonoid yield. The optimized extraction conditions were 80% ethanol, a solid–liquid ratio of 1:60 g/mL, 50 °C, 335 W, and 40 min, yielding 11.005 ± 0.663 mg/g of total flavonoids, which closely matched the model prediction. In HeLa cells, OFE reduced cell viability in a dose- and time-dependent manner, with the most pronounced effect observed at 48 h in both the MTT assay and Calcein-AM/PI staining. Annexin V-FITC/PI analysis showed that apoptotic cell populations increased with escalating OFE exposure. Mechanistically, OFE treatment was associated with reduced p-PI3K and p-AKT levels and increased cleaved caspase-3 expression.

**Discussion:**

These findings establish a practical UAE procedure for the preparation of total flavonoids from *P. physaloides* and provide *in vitro* evidence that OFE exerts cytotoxic effects in HeLa cells by promoting apoptosis-associated responses, potentially through modulation of PI3K/AKT-related survival signaling.

## 1 Introduction

Cervical cancer remains a major cause of cancer-related morbidity and mortality among women worldwide (Caruso et al., 2024). Although vaccination programs and screening strategies have reduced incidence in some regions, therapeutic resistance and tumor recurrence continue to limit long-term clinical outcomes (Hamid et al., 2025). Aberrant activation of pro-survival signaling pathways, particularly the phosphatidylinositol 3-kinase (PI3K)/AKT cascade, is frequently observed in cervical tumors and contributes to sustained proliferation, metabolic adaptation, and resistance to apoptosis (Vara et al., 2004; Shi et al., 2019). Modulation of dysregulated survival signaling therefore represents an important area of investigation in cervical cancer research.

In cervical cancer models, modulation of PI3K/AKT activity has been associated with altered caspase activation and apoptotic progression (Liu et al., 2023). Suppression of AKT phosphorylation in HeLa and related cell lines has been linked to reduced downstream signaling through mTOR and enhanced cleavage of caspase-3 (Raina et al., 2021). In parallel, redox perturbation beyond the buffering capacity of tumor cells has been shown to promote mitochondrial membrane depolarization and intrinsic apoptotic signaling (Pan et al., 2014; Lee et al., 2021). These observations collectively support a functional link between redox balance, PI3K/AKT signaling, and apoptosis regulation in cervical cancer systems.

Natural products have long served as an important source of lead compounds in anticancer drug discovery (Newman and Cragg, 2020). Among plant-derived metabolites, flavonoids constitute one of the most extensively investigated groups of bioactive polyphenols. Extensive experimental studies indicate that flavonoids exhibit a broad range of biological activities relevant to human health, including antioxidant, anti-inflammatory, cardioprotective, and anticancer effects (Panche et al., 2016). In the context of cancer research, many flavonoids have been shown to inhibit tumor cell proliferation through multiple mechanisms, such as regulation of cell-cycle progression, induction of mitochondria-mediated apoptosis, and interference with signaling pathways that control cellular survival and growth (Kopustinskiene et al., 2020). Representative flavonoids including quercetin, luteolin, and kaempferol have been reported to induce G1/S or G2/M cell-cycle arrest and activate caspase-dependent apoptotic responses in various cancer models (Imran et al., 2019; Tang et al., 2020). In addition to their direct effects on tumor cell proliferation, flavonoids are closely associated with the regulation of intracellular redox homeostasis. By modulating cellular levels of reactive oxygen species (ROS) and reinforcing endogenous antioxidant systems, these metabolites may help reduce oxidative DNA damage and thereby limit early tumorigenic events. Interestingly, accumulating evidence also indicates that flavonoids can exert context-dependent pro-oxidant effects in cancer cells. Under certain conditions, such redox perturbations may promote mitochondrial stress and subsequently activate intrinsic apoptotic signaling pathways. Recent pathway-oriented studies further suggest that shifts in ROS dynamics may occur upstream of mitochondrial depolarization and caspase-dependent apoptosis in several cancer systems (Rana et al., 2025; Rana and Mumtaz, 2025). Collectively, these findings indicate that flavonoids can influence tumor cell fate through coordinated regulation of redox balance and survival-associated signaling pathways.

From a structural perspective, flavonoids share a characteristic C6–C3–C6 backbone but exhibit considerable diversity resulting from variations in hydroxylation, methylation, and glycosylation patterns, which ultimately influence their physicochemical properties and biological activities (Stachelska et al., 2025). For example, differences in hydroxylation patterns can markedly alter antioxidant capacity as well as interactions with intracellular signaling networks. Quercetin, which contains multiple hydroxyl groups on the B ring, has frequently been reported to display strong antioxidant activity and to participate in the modulation of signaling pathways associated with tumor cell survival and apoptosis. By contrast, methylated flavonoids often exhibit altered membrane permeability and metabolic stability, which may affect their cellular availability and biological activity (Wolfe and Liu, 2008). Glycosylation represents another important source of flavonoid functional diversity. Although flavonoid glycosides generally possess improved aqueous solubility, they often require enzymatic hydrolysis to release the active aglycone within cells, thereby influencing bioavailability and pharmacological activity (Lai et al., 2025). In addition, several flavonoid subclasses have been reported to affect PI3K/AKT phosphorylation, mitochondrial integrity, and downstream apoptotic signaling in epithelial cancer systems (Zughaibi et al., 2021). Notably, such regulatory effects are frequently described for flavonoid-rich fractions rather than isolated metabolites, suggesting that compositional complexity may contribute to multi-target biological responses (Wagner and Ulrich-Merzenich, 2009). These considerations highlight the importance of investigating flavonoid-enriched botanical extracts within defined cellular contexts.

*Physochlaina physaloides* (L.) G. Don is a botanical drug of the Solanaceae family that has long been used in Mongolian traditional medicine. Phytochemical investigations have revealed that this species contains a wide range of secondary metabolites, including alkaloids, flavonoids, coumarins, and iridoids (Zang et al., 2021). Many of these metabolites exhibit antioxidant or cytotoxic activities. In particular, several metabolites isolated from the roots of *P. physaloides* have been reported to inhibit the proliferation of gastric and liver cancer cells by inducing apoptosis and cell-cycle arrest (Wei et al., 2022), suggesting that this plant may represent a potential source of antitumor bioactive metabolites. Despite these findings, studies focusing specifically on flavonoid-enriched extracts from *P. physaloides* remain relatively scarce. Moreover, few investigations have combined statistically guided extraction optimization with subsequent biological evaluation, and the signaling mechanisms associated with flavonoid-rich fractions in cervical cancer systems are still poorly understood. Another issue is that extraction parameters can substantially influence the phytochemical composition of plant extracts, which may introduce variability in experimental outcomes. However, optimization strategies are not always incorporated into biological studies (Sun et al., 2025). Ultrasound-assisted extraction (UAE) has been increasingly employed for the recovery of polyphenolic compounds because acoustic cavitation enhances solvent penetration and mass transfer while operating under relatively mild thermal conditions (Özdemir et al., 2025). Compared with conventional extraction techniques such as reflux or maceration, UAE can reduce extraction time and solvent usage while improving extraction efficiency, thereby helping preserve structurally sensitive flavonoid metabolites (Zhang et al., 2018). Nevertheless, the efficiency of UAE is strongly affected by operational variables, including solvent composition, ultrasound power, and extraction duration. Without appropriate optimization, variations in these parameters may lead to differences in phytochemical composition and compromise experimental consistency (Chemat et al., 2017). Therefore, integrating statistically guided extraction optimization with subsequent biological assessment may provide a practical approach for exploring the biological activity of flavonoid-enriched extracts.

The present study aimed to verify the anti-cervical-cancer activity of the total flavonoid fraction from *P. physaloides* using *in vitro* cell-based assays and to provide initial clues to its molecular regulatory mechanisms. Accordingly, this work pursued two complementary objectives. First, we aimed to establish a statistically optimized ultrasound-assisted extraction (UAE) protocol for obtaining a flavonoid-enriched fraction from *P. physaloides* using Plackett–Burman screening and Box–Behnken response surface methodology. Second, we evaluated whether the optimized extract influences apoptosis-associated signaling in a cervical cancer cell model, with particular attention to PI3K/AKT phosphorylation and caspase activation. By integrating extraction optimization with pathway-oriented *in vitro* assessment, this study provides methodological support for the systematic evaluation of flavonoid-enriched botanical fractions and offers preliminary biological evidence that may facilitate future chemical characterization, *in vivo* validation, and the potential development of plant-derived anticancer agents.

## 2 Materials and methods

### 2.1 Plant materials and reagents

To assess site-to-site variation, roots of *P. physaloides* were sampled from two locations in Inner Mongolia, China. One batch of roots (Sample A) was collected in June 2025 at Daqingshan (41°00′42″N, 111°50′46″E; 1,537 m above sea level). A second batch of roots (Sample B) was obtained in July 2025 from Xilingol (43°51′20″N, 113°38′23″E; 1,041 m above sea level). The species was authenticated by Prof. Jie Xu (Inner Mongolia Normal University), and voucher specimens (Sun ZY 20250608 (NMTC) and Sun ZY 20250724 (NMTC)) were deposited in the university herbarium. Plant materials were air-dried in the shade, milled using a multifunction grinder (XA-1; Surui Instrument Co., Ltd., China), passed through a 100-mesh sieve, and kept in desiccators under ambient conditions prior to extraction. This study was revised with reference to the ConPhyMP tool and the Four Pillars of Best Practice in Ethnopharmacology; the completed ConPhyMP checklist/tables are provided as Supplementary Material.

Rutin (≥98%, analytical reference standard) was sourced from Shandong Keyuan Biochemical Co., Ltd. Unless specified otherwise, routine analytical reagents were provided by Inner Mongolia Manhui Trading Co., Ltd. (Inner Mongolia, China); this included anhydrous ethanol as well as sodium nitrite, aluminum nitrate, and sodium hydroxide. HeLa cells were obtained from Servicebio (Wuhan, China). Cell culture employed Gibco DMEM as the base medium with 10% fetal bovine serum and 1% penicillin–streptomycin. For viability-related assays, MTT (Aladdin, Shanghai, China) and a Calcein-AM/PI live/dead staining kit (Solarbio, Beijing, China) were used. Apoptosis was assessed using an Annexin V-FITC/PI detection kit (Beyotime, Shanghai, China). Primary and HRP-conjugated secondary antibodies for immunoblotting were supplied by Shenyang Wanlei Biotechnology (Shenyang, China).

### 2.2 Instruments and equipment

Equipment used in this work comprised an analytical balance (PX84ZH; 0.1 mg; Ohaus, Changzhou, China), a thermostatic drying oven (101-3AB; Taisite, Tianjin, China), and a UV–Vis spectrophotometer (UV-1800PC; Aoyi, Shanghai, China). A thermostatic water bath (HHS-21-6; Boxun, Shanghai, China), an ultrasonic cleaner (SB25-12DTD; Ningbo Xinzhi, Ningbo, China), a microplate reader (H1M; Drei Biotechnology, Guangzhou, China), a refrigerated high-speed centrifuge (H17.5R; Shanghai Luxiangyi, Shanghai, China), a fluorescence microscope (IX53; Olympus, Japan), and a flow cytometer (CytoFLEX S; Beckman, American) were also used.

### 2.3 Ultrasound-assisted extraction (UAE) and total flavonoid quantification

#### 2.3.1 Standard curve preparation

Total flavonoids were measured by an aluminum–reagent color assay referenced to rutin (Jia et al., 1999). Rutin standards were prepared from a 0.2 mg/mL solution in 60% ethanol (working range: 10–60 μg/mL). Color development proceeded under the following timing scheme: NaNO_2_ 5% (6 min) → Al(NO_3_)_3_ 10% (6 min) → NaOH 4% (15 min). Absorbance was read at 510 nm, and the calibration fit was *A* = 0.0108*C* − 0.0155 (*R* ^2^ = 0.9922) (*A*, absorbance; *C*, rutin concentration in μg/mL).

#### 2.3.2 Sample extraction and assay

Air-dried root material was milled and weighed (2.0 g) into a conical flask. UAE was carried out with ethanol, with solvent volume and concentration assigned according to the experimental design, and sonication was performed at the preset time, temperature, and power. The suspension was then clarified by filtration followed by centrifugation (5,600 rpm, 10 min). The resulting supernatant was collected and, when necessary, brought to the appropriate range with 70% ethanol before analysis using the colorimetric procedure in Section 2.3.1. Total flavonoids were expressed as *P* (mg/g) and calculated as:

$$P=\left(c\times V_{1}\times V\right)\;/\;\left(m\times V_{2}\right),$$

Variables: *c* (µg/mL, from the calibration curve), *V* (mL, total extract volume), *V*_*1*_ (mL, total assay volume), *V*_*2*_ (mL, sample volume used), and *m* (g, sample mass) (Luo et al., 2010). Each extraction and determination was conducted in triplicate.

### 2.4 Experimental design and statistical analysis

#### 2.4.1 Single-factor experiments

Initial single-parameter tests were used to bracket the operating windows: sonication time (20–60 min), temperature (30 °C–50 °C), power (250–450 W), ethanol strength (50%–90%), and solvent-to-solid ratio (10–60 mL/g). For the subsequent design, each factor was centered around the level associated with the highest total flavonoid yield in the screening runs.

#### 2.4.2 Plackett-burman (PB) design

Factor screening was performed with a 12-run Plackett–Burman (PB) design constructed in Design-Expert 13. The five candidate variables were evaluated at two coded settings (−1 for low and +1 for high; Table 1). Model coefficients and the corresponding ANOVA outputs were obtained directly from the Design-Expert analysis module.

**TABLE 1:** Factors and levels of PB experiments.

Levels	A (ultrasonic time/min)	B (ultrasonic temperature/°C)	C (ultrasonic power/W)	D (ethanol volume fraction/%)	E (solid–liquid ratio/(g/mL))
Low (−1)	30	40	250	70	1:40
High (+1)	50	50	350	90	1:60

Based on the PB design results, the three most significant factors were selected for a steepest-ascent experiment to approach the optimal region rapidly.

#### 2.4.3 Optimization with box-behnken design and model verification

To explore interactions among the three key variables, a Box–Behnken response-surface scheme was implemented with three levels per factor (Table 2). The response data were described with a quadratic equation, whose adequacy was evaluated by ANOVA in Design-Expert. Three independent confirmation experiments subsequently checked the predicted optimum.

**TABLE 2:** Variables and their levels in the Box–Behnken design.

Factor	Level		
	−1	0	1
X_1_ solid–liquid ratio (g/mL)	1:50	1:55	1:60
X_2_ ultrasonic temperature (°C)	45	47.5	50
X_3_ ultrasonic power (W)	300	325	350

### 2.5 Preparation of the optimized flavonoid extract (OFE) for bioassay

To generate sufficient material for downstream bioassays, a larger-scale preparation was performed using 50 g of powdered *P. physaloides* root (Sample B). The powder was first washed with petroleum ether (b.p. 30 °C–60 °C) to remove nonpolar metabolites, and the residue was then dried. UAE then extracted flavonoids under the optimized settings established in this study. The pooled extracts were clarified by filtration, the solvent was removed by rotary evaporation under vacuum, and the concentrate was further cleaned up on a D-101 macroporous resin column. Elution was performed using stepwise ethanol–water mixtures, and the flavonoid-enriched fractions were combined and concentrated. The resulting fraction was freeze-dried (GIPP-02FDA vacuum lyophilizer; Shanghai GIPP Electronic Technology Co., Ltd., Shanghai, China) to yield a dry powder, termed the Optimized Flavonoid Extract (OFE). OFE aliquots were stored at −20 °C in light-protective containers until cell-based experiments.

### 2.6 Assessment of cytotoxic activity in HeLa cervical cancer cells

#### 2.6.1 Cell culture and OFE treatment (HeLa and H8)

HeLa cells and non-transformed human cervical epithelial cells (H8; Shanghai Yaji Biotechnology, Shanghai, China; catalog no. YS1628C) were maintained in a humidified incubator at 37 °C with 5% CO_2_. Both cell lines were cultured in high-glucose DMEM supplemented with 10% fetal bovine serum (FBS; Gibco, USA) and 1% penicillin–streptomycin (Sigma-Aldrich, USA). The optimized flavonoid extract (OFE) was prepared as a 100 mg/mL stock solution in dimethyl sulfoxide (DMSO; Sigma-Aldrich, USA), aliquoted, and stored at −20 °C. For each experiment, an aliquot was thawed and diluted into complete medium immediately before use. Vehicle was matched across all groups, and the final DMSO concentration was kept at 0.1% (*v/v*) or below. Vehicle controls showed no detectable effect on cell viability.

#### 2.6.2 Cell viability assay (MTT)

Antiproliferative effects of OFE were quantified using an MTT colorimetric readout (Van Meerloo et al., 2011). HeLa cells were plated in 96-well plates (8 × 10^3^ cells/well) and allowed to attach for 24 h, after which OFE was applied at 12.5–50 μg/mL for 24–72 h (final DMSO ≤0.1% in all wells). MTT reagent (20 μL per well; 5 mg/mL in PBS) was then introduced, and incubation continued for 4 h at 37 °C. Culture medium was removed, formazan was dissolved, and absorbance was read at 490 nm using a microplate reader (H1M; Drei Biotechnology Co., Ltd., Guangzhou, China). H8 cells were assayed in parallel using the same seeding density, OFE concentrations, and treatment durations. To evaluate whether OFE directly affected the MTT colorimetric readout, parallel cell-free control wells were included. These wells contained culture medium, OFE at the indicated concentrations, and MTT reagent, but no cells. After incubation, the wells were processed in the same manner as the experimental wells, and absorbance was measured at 490 nm to assess potential interference of the extract with the assay signal itself. Viability and growth-inhibition were computed using the equations below:

$$Cell\;viability\;\left(\%\right)=\frac{A_{490,treated}-{\bar{A}}_{490,no-cell}}{{\bar{A}}_{490,control}-{\bar{A}}_{490,no-cell}}\times 100.$$

#### 2.6.3 Morphological assessment by live/Dead staining

For live/dead visualization, cells were assigned to a vehicle control and three OFE concentrations, and all groups were incubated for 48 h. Cells were briefly rinsed with PBS and stained with Calcein-AM (2 μM) and PI (4 μM) for 30 min at 37 °C under light-protected conditions (Wang et al., 2025). After a quick PBS wash, fluorescence images were captured on an inverted microscope. Calcein-positive cells were interpreted as viable, whereas PI-positive cells were treated as membrane-compromised.

#### 2.6.4 Flow cytometric analysis of apoptosis

Apoptosis was assessed by Annexin V-FITC/PI labeling and flow cytometric analysis (Vermes et al., 1995). After 48 h of exposure, cells were harvested and normalized to a uniform density. Staining was performed according to the kit instructions using the following workflow: 195 μL binding buffer per sample, Annexin V-FITC 5 μL (10 min, room temperature, light-protected), PI 10 μL (20 μg/mL; 5 min on ice), then an additional 300 μL binding buffer immediately prior to acquisition. Fluorescence was recorded in the FITC (Ex 488 nm/Em 530 nm) and PI (Ex 535 nm/Em 617 nm) channels. Populations were assigned by quadrant gating on FITC–PI dot plots (viable, early apoptosis, late apoptosis, and PI-only/necrotic) (Alshehade et al., 2024).

#### 2.6.5 Western blotting

Cells were lysed on ice in pre-chilled RIPA buffer, and clarified lysates were obtained by centrifugation (12,000 rpm, 1 min). Protein content was determined by BCA (Bainor et al., 2011). Equal amounts of protein were resolved by SDS–PAGE and transferred to nitrocellulose membranes. Blocking was performed for 2 h in 5% (*w/v*) non-fat milk prepared in TBS containing 0.1% Tween-20. Membranes were incubated with primary antibodies at 4 °C overnight, washed with TBST (5 times), and then incubated with HRP-conjugated secondary antibodies for 1 h at room temperature. Signals were developed using an ECL substrate and quantified in ImageJ; band intensities were normalized to β-actin. Experiments were conducted in three independent repeats, with low/intermediate/high doses matching those used in the MTT assay for both OFE and the positive control.

### 2.7 Statistical analysis

For each assay, data were obtained from ≥3 independent repeats and are presented as mean ± SD. Statistical analyses were conducted in GraphPad Prism (v9.5.1). For multiple-group comparisons, a one-way ANOVA with Dunnett *post hoc* testing against the control group was performed; *p* < 0.05 (two-sided) was considered significant. Fluorescence images were arranged in Adobe Photoshop (CC 2019) for layout only, without altering raw signals.

## 3 Results

### 3.1 Selection of plant material based on flavonoid content

**FIGURE 1:**
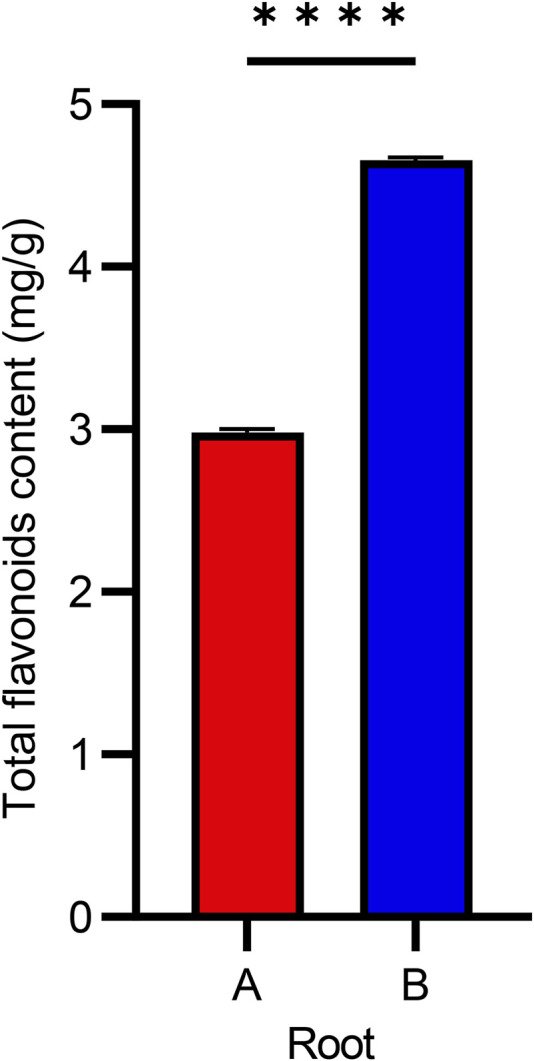
Comparison of Total Flavonoid Content in Two Batches of *Physochlaina physaloides* (L.) G. Don Samples. **(A)** Roots collected from the Daqingshan region; **(B)** Roots collected from the Xilingol League region.

The total flavonoid content of the root samples of *P. physaloides* differed notably between the two collection sites, with Sample B from Xilingol exhibiting a higher flavonoid yield than Sample A from Daqingshan (Figure 1). The higher flavonoid content in Sample B may reflect site-specific environmental differences. Xilingol generally experiences lower UV-related stress and less pronounced diurnal temperature variation than higher-altitude areas, factors that have been linked to shifts in flavonoid biosynthesis in plants (Dotto and Casati, 2017). Soil moisture and nutrient availability may also contribute, as edaphic conditions are known to shape secondary-metabolite profiles (Yang et al., 2018; Tang et al., 2024).

### 3.2 Single-factor experiment analysis

**FIGURE 2:**
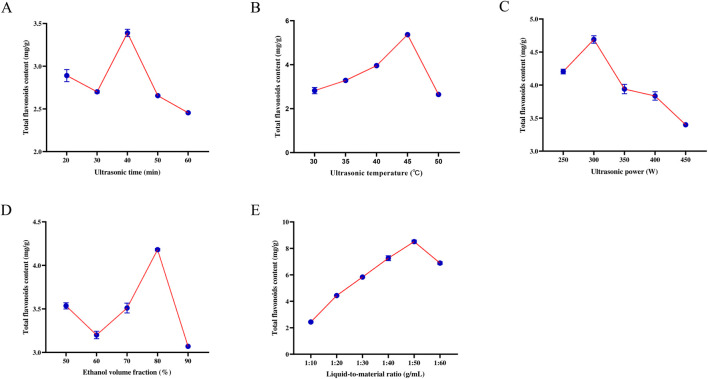
Effects of Ultrasonic time **(A)** Ultrasonic temperature **(B)** Ultrasonic power **(C)** Ethanol volume fraction **(D)** and Solid–liquid ratio **(E)** on total flavonoid content.

The influence of ultrasonic time, temperature, power, ethanol concentration, and solid–liquid ratio on total flavonoid yield from *P. physaloides* is shown in Figure 2. Yield increased as the sonication time was extended from 20 to 40 min, but decreased when the time was further prolonged (Figure 2A). Increasing the temperature enhanced extraction up to 45 °C, whereas a marked reduction was observed at 50 °C (Figure 2B). For ultrasonic power, the highest yield was obtained at 300 W, and higher power was associated with lower yield (Figure 2C). Ethanol concentration produced the highest yield at 80% (Figure 2D). For the solid–liquid ratio, yield increased up to 1:50 g/mL and then declined at 1:60 g/mL (Figure 2E). Based on these trends, the preliminary optimal conditions for subsequent experimental screening were established as follows: ultrasonic time of 40 min, temperature of 45 °C, power of 300 W, ethanol concentration of 80%, and solid-liquid ratio of 1:50 g/mL.

### 3.3 Screening of significant factors via plackett-burman design

**FIGURE 3:**
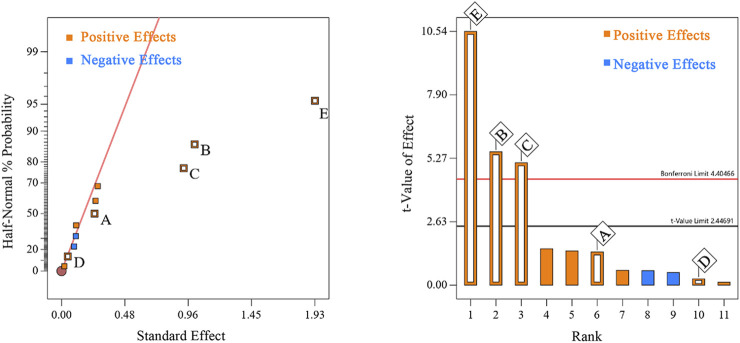
Half-Normal Plot and Pareto Chart. **(A)** Ultrasonic time; **(B)** Ultrasonic temperature; **(C)** Ultrasonic power; **(D)** Ethanol volume fraction; **(E)** Solid–liquid ratio.

A 12-run PB design was executed to screen the five factors. The experimental design and corresponding responses are shown in Table 3. ANOVA of the PB model (Table 4) indicated that the model was highly significant (*p* = 0.0003). The half-normal probability plot and Pareto chart (Figure 3), which are widely recommended for visually identifying statistically significant effects in screening designs (Mašković et al., 2010), clearly indicated three factors with significant positive influences (*p* < 0.05) on flavonoid yield: solid-liquid ratio (E), ultrasonic temperature (B), and ultrasonic power (C). In contrast, factors A (ultrasonic time) and D (ethanol concentration) showed no statistically significant effects within the tested ranges. The fitted first-order model was:*Y* = 5.80 + 0.1262*A*+ 0.5073*B*+ 0.4650*C* + 0.0237*D* + 0.9648*E* (*R* ^2^ = 0.9658).

**TABLE 3:** Design and results of PB experiments.

Experimental run	Coded symbols					Total flavonoid content (mg/g)
	A	B	C	D	E	
1	1	1	−1	1	1	6.986
2	−1	1	1	−1	1	8.000
3	1	−1	1	1	−1	5.213
4	−1	1	−1	1	1	6.625
5	−1	−1	1	−1	1	6.444
6	−1	−1	−1	1	−1	3.778
7	1	−1	−1	−1	1	5.611
8	1	1	−1	−1	−1	5.231
9	1	1	1	−1	−1	5.593
10	−1	1	1	1	−1	5.417
11	1	−1	1	1	1	6.931
12	−1	−1	−1	−1	−1	3.787

**TABLE 4:** Variance analysis of PB experiments.

Source	Sum of squares	df	Mean square	*F*-value	*P*-value
Model	17.05	5	3.41	33.91	0.0003
A (ultrasonic time)	0.1910	1	0.1910	1.90	0.2173
B (ultrasonic temperature)	3.09	1	3.09	30.71	0.0015
C (ultrasonic power)	2.59	1	2.59	25.80	0.0023
D (ethanol volume fraction)	0.0067	1	0.0067	0.0668	0.8046
E (solid–liquid ratio)	11.17	1	11.17	111.08	<0.0001
Residual	0.6034	6	0.1006	​	​
Cor total	17.66	11	​	​	​
*R* ^*2*^	​	​	​	​	0.9658
*R^2^* adj	​	​	​	​	0.9373
*R^2^* pred	​	​	​	2	0.8633

df, Degree of freedom; *R* ^*2*^ and *R^2^* adj are calculated for the reduced model; *:*P* < 0.05, **P < 0.01, ***P < 0.001.

The three significant variables (E, B, and C) were explored by a steepest-ascent search while holding ethanol concentration at 80% and sonication time at 40 min. The stepwise settings and corresponding responses are summarized in Table 5. Flavonoid yield increased across the ascent steps and peaked at 11.081 mg/g at step 4 (solid–liquid ratio 1:55, 47.5 °C, 325 W). This condition was therefore used as the center point for the subsequent RSM study.

**TABLE 5:** Design and results of the steepest climb test.

No.	Pace	Solid–liquid ratio (g/mL)	Ultrasonic temperature (°C)	Ultrasonic power (W)	Total flavonoid content (mg/g)
1	0	1:40	40	250	9.830
2	0+1Δ	1:45	42.5	275	9.726
3	0+2Δ	1:50	45	300	10.127
4	0+3Δ	1:55	47.5	325	11.081
5	0+4Δ	1:60	50	350	10.801

Δ is a step unit.

### 3.4 Response surface–guided optimization of UAE parameters

A Box–Behnken response-surface scheme (BBD) was adopted, comprising 17 experimental runs with a center-point replicate. The corresponding design matrix and observed responses are summarized in Table 6. The experimental data were described using a quadratic (second-order) regression model: *R* = 11 + 0.3393*X*_1_ + 0.2374*X*_2_ + 0.1299*X*_3_ − 0.0745*X*_1_ *X*_2_ + 0.0935*X*_1_ *X*_3_ + 0.0902*X*_2_ *X*_3_ − 0.4351*X*_1_^2^ − 0.1153*X*_2_^2^ − 0.4528*X*_3_^2^, in which *X*_1_, *X*_2_, and *X*_3_ denote the coded levels of the solid–liquid ratio, ultrasonic temperature, and ultrasonic power, respectively.

**TABLE 6:** Response surface test design and results.

No.	X_1_	X_2_	X_3_	Total flavonoid content (mg/g)
1	−1	−1	0	9.811
2	1	−1	0	10.625
3	−1	1	0	10.428
4	1	1	0	10.944
5	−1	0	−1	9.722
6	1	0	−1	10.227
7	−1	0	1	9.815
8	1	0	1	10.694
9	0	−1	−1	10.164
10	0	1	−1	10.465
11	0	−1	1	10.223
12	0	1	1	10.885
13	0	0	0	11.076
14	0	0	0	11.064
15	0	0	0	10.885
16	0	0	0	10.962
17	0	0	0	11.025

**FIGURE 4:**
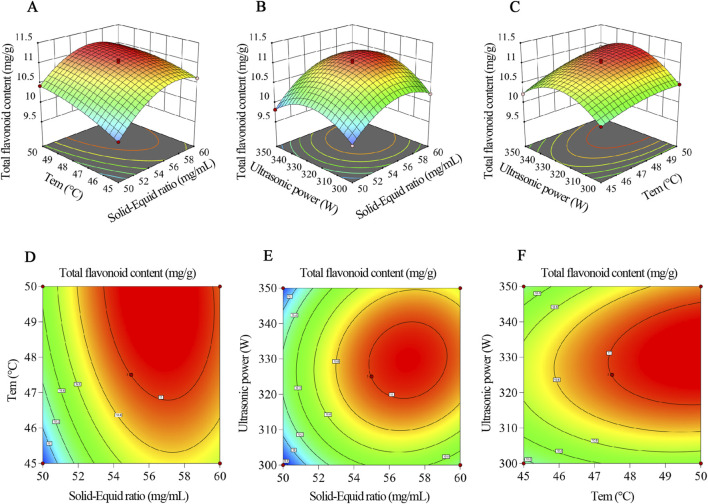
Response surface analysis of total flavonoid content from *Physochlaina physaloides* (L.) G. Don. **(A–C)** Three-dimensional response surface plots illustrating the interactive effects of **(A)** solid–liquid ratio and ultrasonic temperature, **(B)** solid–liquid ratio and ultrasonic power, and **(C)** ultrasonic temperature and ultrasonic power on total flavonoid content. **(D–F)** Corresponding two-dimensional contour plots of panels **(A–C)**, respectively. In each plot, the third variable was maintained at its central level.

ANOVA for the quadratic model (Table 7) confirmed its high significance (*p* < 0.0001) and excellent fit (*R* ^2^ = 0.9924, Adj-*R* ^2^ = 0.9827). All linear terms (*X*_1_, *X*_2_, *X*_3_), interaction terms (*X*_1_ *X*_3_, *X*_2_ *X*_3_), and quadratic terms (*X*_1_^2^, *X*_2_^2^, *X*_3_^2^) were significant (*p* < 0.05). The *F*-values indicated that the factors’ influence on the extraction yield followed the order: solid-liquid ratio > ultrasonic temperature > ultrasonic power. The three-dimensional response surface plots illustrating these interactions are shown in Figure 4.

**TABLE 7:** Analysis of variance of a regression equation.

Source	Sum of squares	df	Mean square	*F*-value	*P*-value	Significance
Model	3.46	9	0.3848	101.99	<0.0001	**
*X*_1_	0.9207	1	0.9207	244.01	<0.0001	**
*X*_2_	0.4508	1	0.4508	119.47	<0.0001	**
*X*_3_	0.1349	1	0.1349	35.76	0.0006	**
*X*_1_ *X*_2_	0.0222	1	0.0222	5.88	0.0457	*
*X*_1_ *X*_3_	0.0350	1	0.0350	9.27	0.0187	*
*X*_2_ *X*_3_	0.0326	1	0.0326	8.63	0.0218	*
*X*_1_^2^	0.7970	1	0.7970	211.23	<0.0001	**
*X*_2_^2^	0.0560	1	0.0560	14.84	0.0063	**
*X*_3_^2^	0.8634	1	0.8634	228.81	<0.0001	**
Residual	0.0264	7	0.0038	​	​	​
Lack of fit	0.0013	3	0.0004	0.0677	0.9742	Not significant
Pure error	0.0251	4	0.0063	​	​	​
Cor total	3.49	16	​	​	​	​
*R* ^*2*^	​	​	​	​	0.9924	​
*R^2^* adj	​	​	​	​	0.9827	​
*R^2^* pred	​	​	​	​	0.9829	​

X_1_, Solid–liquid ratio, X_2_, Ultrasonic temperature; X_3_, Ultrasonic power; df, Degree of freedom; *R* ^*2*^.

*R^2^* adj and *R^2^* pred are calculated for the reduced model; *:*P* < 0.05, **P < 0.01, ***P < 0.001.

The model predicted the optimal extraction conditions to be: a solid-to-liquid ratio of 1:57.36 g/mL, an ultrasonic temperature of 48.60 °C, and an ultrasonic power of 338.64 W. Under these conditions, the predicted maximum total flavonoid content was 11.114 mg/g.

A slight adjustment was made to the model-derived optimum to make the procedure easier to implement in routine practice. The final operating parameters were set to a solid–liquid ratio of 1:60 (g/mL), a temperature of 50 °C, and an ultrasonic power of 335 W. Using these settings, three independent validation runs gave a mean total flavonoid content of 11.005 ± 0.663 mg/g (mean ± SD), consistent with the predicted response. The dispersion across repeats was limited (CV = 5.97%), suggesting that the optimized extraction protocol is stable and readily applicable.

### 3.5 Cytotoxic effects of the optimized flavonoid extract on HeLa cells and H8

**FIGURE 5:**
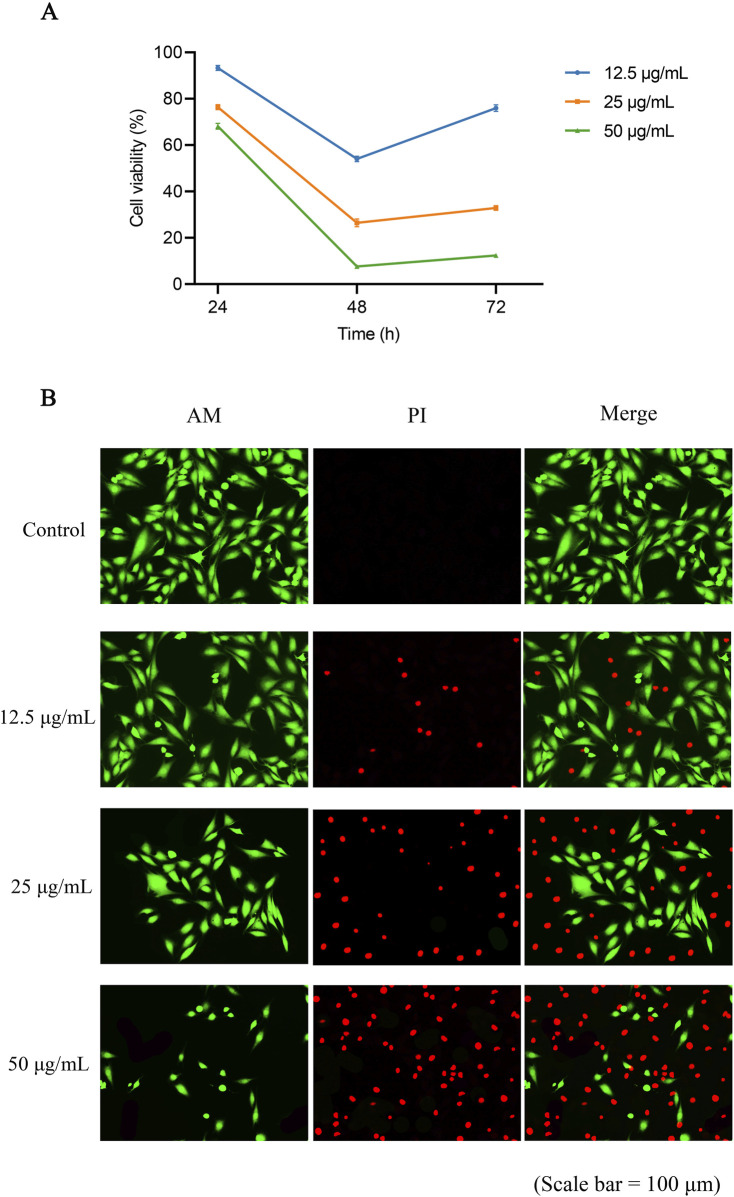
Cytotoxic effects of the optimized flavonoid extract (OFE) on HeLa cells assessed by MTT assay and Calcein-AM/PI live/dead staining. **(A)** Cell viability (%) of HeLa cells measured by the MTT assay after treatment with OFE at 12.5, 25, and 50 μg/mL for 24, 48, and 72 h. Viability values were corrected using the corresponding no-cell control. **(B)** Representative fluorescence images of Calcein-AM/PI live/dead staining after 48 h of OFE treatment. Live cells are shown by green Calcein-AM fluorescence, whereas dead cells with compromised membrane integrity are indicated by red propidium iodide (PI) staining. Scale bar = 100 μm.

OFE treatment significantly reduced HeLa cell viability in a concentration-dependent manner, with the strongest reduction observed at 48 h, as determined by the MTT assay after correction with the corresponding no-cell control (Figure 5A). At 24 h, relative viability decreased to 93.3%, 76.4%, and 68.1% at 12.5, 25, and 50 μg/mL, respectively. At 48 h, viability further declined to 54.0%, 26.5%, and 7.6%. At 72 h, OFE maintained pronounced cytotoxic activity, with viability values of 76.0%, 32.9%, and 12.3% at the same concentrations. One-way ANOVA showed significant treatment effects at all three time points (24 h: *p* < 0.0001; 48 h: *p* < 0.0001; 72 h: *p* < 0.0001), and Dunnett’s multiple comparisons test confirmed significant reductions in viability for all OFE-treated groups versus control at each time point (all adjusted *p* < 0.0001).

Compared with HeLa cells, H8 cells showed only minimal viability reduction across 24–72 h at the tested OFE concentrations, suggesting preferential sensitivity of the cancer cell model. Detailed H8 results are provided in the Supplementary Material (Supplementary Table S1). Live/dead staining performed at 48 h only (Figure 5B) further supported the cytotoxic effects observed in the MTT assay. Control cells exhibited strong Calcein-AM fluorescence with minimal PI staining, indicating preserved membrane integrity. In OFE-treated groups, PI-positive cells progressively increased with rising concentrations, accompanied by a visible reduction in Calcein-AM-positive cells. This concentration-dependent shift was most pronounced at 50 μg/mL, consistent with the marked reduction in viability detected in the 48 h MTT analysis.

### 3.6 Flow cytometric analysis of apoptosis and PI-positive populations

**FIGURE 6:**
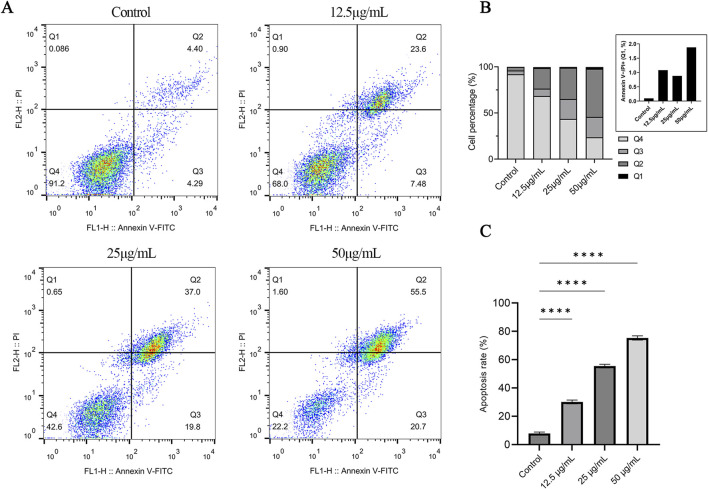
Annexin V-FITC/PI flow cytometry analysis of OFE-induced apoptosis in HeLa cells (48 h). **(A)** Representative dot plots of Annexin V-FITC (x-axis) versus PI (y-axis) following treatment with OFE (12.5, 25, and 50 μg/mL) or vehicle control for 48 h; quadrant percentages are indicated. Quadrants were defined as: Q4, Annexin V-/PI- (live); Q3, Annexin V+/PI- (early apoptosis); Q2, Annexin V+/PI+ (late apoptosis/PI-positive, consistent with late apoptosis and/or secondary necrosis); Q1, Annexin V-/PI+ (necrosis/mechanical damage). **(B)** Quadrant distribution across groups shown as a 100% stacked bar chart; inset shows an expanded view of Q1 (Annexin V^−^/PI^+^) to facilitate visualization of the low-frequency necrotic fraction. **(C)** Total apoptosis rate calculated as Annexin V–positive fractions (Q2 + Q3) (mean ± SD, n = 3). Statistical analysis: one-way ANOVA followed by Dunnett’s multiple comparisons test versus control. ****p < 0.0001.

Annexin V-FITC/PI flow cytometry performed after 48 h of OFE exposure showed a clear dose-related redistribution of cells from the viable quadrant (Annexin V^−^/PI^−^) toward Annexin V–positive populations (Figure 6A). Quantitatively, the total Annexin V–positive fraction (Q2 + Q3) rose from 7.89% ± 0.97% in the control group to 30.13% ± 1.35%, 55.42% ± 1.28%, and 75.28% ± 1.52% at 12.5, 25, and 50 μg/mL, respectively (Figure 6C). Group differences were significant by one-way ANOVA (*p* < 0.0001), and Dunnett’s *post hoc* testing confirmed that each OFE-treated group differed from control (adjusted *p* < 0.0001).

To address PI-positive/necrotic populations separately, quadrant distributions were summarized across doses (Figure 6B). The Annexin V^−^/PI^+^ population (Q1; necrosis/mechanical damage) remained a minor fraction throughout the experiment, increasing only slightly from 0.10% ± 0.03% (control) to 1.08% ± 0.19%, 0.88% ± 0.21%, and 1.88% ± 0.25% with increasing OFE concentration. In contrast, the Annexin V^+^/PI^+^ population (Q2; late apoptosis) increased markedly from 3.85% ± 0.50% to 22.20% ± 2.82%, 33.86% ± 3.43%, and 52.20% ± 3.29%, while early apoptotic cells (Q3; Annexin V^+^/PI^−^) increased from 4.05% ± 0.57% to 7.93% ± 1.53%, 21.56% ± 2.22%, and 22.07% ± 2.20%. Consistent with these shifts, the viable fraction (Q4) declined from 91.97% ± 1.00% to 68.48% ± 1.67%, 43.56% ± 1.13%, and 23.63% ± 1.36% across the same dose range. The PI-positive increase at high dose was mainly driven by Q2, while Q1 remained a minor fraction.

### 3.7 OFE suppresses PI3K/AKT signaling and promotes caspase-3 activation (western blotting)

**FIGURE 7:**
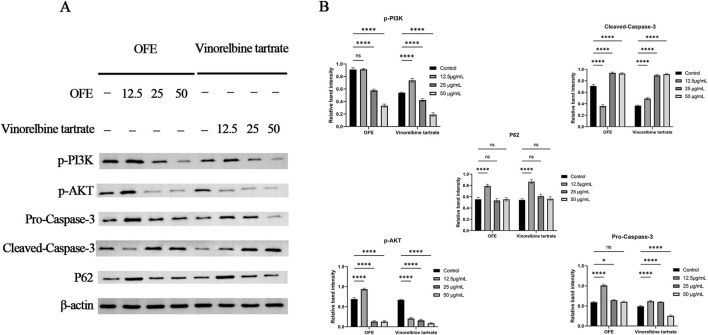
Western blot analysis of PI3K/AKT signaling and apoptosis-related proteins after treatment with OFE or vinorelbine tartrate. **(A)** Representative Western blot bands showing the levels of p-PI3K, p-AKT, pro-caspase-3, cleaved-caspase-3 and p62, with β-actin as the loading control, in cells treated with OFE from *Physochlaina physaloides* (L.) G. Don (OFE) or vinorelbine tartrate (positive-control drug) at the indicated concentrations. **(B)** Densitometric quantification of band intensities normalized to β-actin and expressed relative to the corresponding vehicle control. Data are presented as mean ± SD from n = 3 independent experiments. Statistical analysis was performed using one-way ANOVA followed by Dunnett’s multiple comparisons test versus the vehicle control. *P < 0.05, **P < 0.01, ***P < 0.001, ****P < 0.0001; ns, not significant.

Given that constitutive activation of the PI3K/AKT pathway is common in cervical cancer and promotes cell survival and resistance to apoptosis, we selected PI3K/AKT phosphorylation as a readout of pro-survival signaling. In parallel, caspase-3 cleavage was assessed as a canonical marker of apoptotic execution downstream of survival pathway attenuation (Altomare and Testa, 2005; Galluzzi et al., 2018). As shown in Figure 7A, Western blot analysis demonstrated that OFE treatment was associated with a concentration-dependent reduction in PI3K/AKT phosphorylation. Compared with vehicle control, p-PI3K and p-AKT band intensities progressively decreased at 25 and 50 μg/mL. In contrast, cleaved caspase-3 levels increased at intermediate and high concentrations, whereas pro-caspase-3 exhibited comparatively modest variation across doses.

Densitometric quantification of protein expression (Figure 7B) was performed by normalizing each target protein to β-actin and expressing values relative to vehicle control (set to 1). One-way ANOVA revealed a significant effect of treatment on p-PI3K, p-AKT, and cleaved caspase-3 expression (*p* < 0.001). Post hoc Dunnett’s multiple comparisons test demonstrated that 25 and 50 μg/mL significantly reduced p-PI3K and p-AKT levels and significantly increased cleaved caspase-3 relative to control (adjusted *p* < 0.05–0.0001 as indicated in the figure). In contrast, pro-caspase-3 did not display a consistent dose-dependent pattern, and p62 levels also did not display a consistent dose-dependent pattern across the tested concentrations. The positive-control drug vinorelbine tartrate produced changes in the same direction, including reduced PI3K/AKT phosphorylation and increased caspase-3 cleavage, supporting the internal validity of the assay.

## 4 Discussion

Therapeutic resistance and recurrence remain major barriers to durable control in cervical cancer, and survival-pathway rewiring is a recurring feature of aggressive disease. Within this landscape, flavonoid-rich botanical preparations continue to be explored because polyphenolic metabolites can perturb stress handling, survival signaling, and apoptosis-associated cascades. Yet two practical obstacles often limit interpretability: extract composition is sensitive to processing conditions, and phenotypic outcomes are frequently reported without pathway context or selectivity comparisons to non-transformed epithelial cells.

In this study, we combined extraction optimization with pathway-oriented cellular evaluation to characterize a flavonoid-enriched fraction from *Physochlaina physaloides* (L.) G. Don. Using a DOE strategy (Plackett–Burman screening followed by Box–Behnken response surface methodology), we defined an operating window for ultrasound-assisted extraction and generated OFE for downstream assays. In HeLa cells, OFE reduced viability and increased Annexin V–positive fractions, accompanied by reduced PI3K/AKT phosphorylation and increased caspase-3 cleavage. Under the same exposure schedule, non-transformed H8 cervical epithelial cells maintained comparatively higher viability, suggesting that the growth-inhibitory effect is more pronounced in the cancer cell context within the tested range. Together, these results outline a coherent *in vitro* phenotype that links cellular outcomes to survival- and apoptosis-associated signaling readouts.

Flavonoid-rich fractions from Solanaceae species have previously been reported to suppress HeLa cell growth, supporting the broader view that Solanaceae-derived polyphenolic mixtures can exert measurable anti-proliferative effects in cervical cancer models. For example, extracts from *Physalis peruviana* and *Physalis angulata* L. showed cytotoxic activity in comparable concentration windows (Mier-Giraldo et al., 2017; Pillai et al., 2022), and *Solanum nigrum*–derived metabolites have been associated with anti-HeLa activity together with down-modulation of pro-survival signaling (Nawaz et al., 2021). Collectively, these studies suggest that growth inhibition by Solanaceae flavonoid-rich preparations is reproducible across species, while the signaling context and mechanistic anchoring can differ with composition and experimental design.

In contrast, the present work places a flavonoid-enriched fraction from the comparatively under-characterized species *P. physaloides* into this landscape with pathway-linked evidence. Rather than relying on viability as a standalone endpoint, we coupled apoptosis phenotypes with PI3K/AKT phosphorylation changes and caspase-3 cleavage, providing an interpretable signaling framework consistent with suppression of a survival axis alongside apoptotic execution. The inclusion of H8 cells further adds an initial selectivity context, indicating weaker cytotoxicity in a non-transformed cervical epithelial model under the same exposure schedule. Because OFE is a mixture, these findings are best interpreted as an association between extract exposure and pathway state rather than direct engagement of a single molecular target. Consistent with this interpretation, UHPLC–MS/MS profiling further indicated that OFE remained a chemically complex preparation with multiple preliminarily annotated metabolites rather than a single defined entity (Supplementary Figure S1; Supplementary Table S2). Therefore, the present data support an extract-level, pathway-associated phenotype, but do not identify the specific metabolites responsible for the observed activity.

Mechanistically, PI3K/AKT signaling is a well-established survival axis in cancer cells, and reduced phosphorylation is compatible with lowering the threshold for apoptotic execution (Abotaleb et al., 2018). The observed increase in cleaved caspase-3 aligns with this directionality. An important question is whether PI3K/AKT attenuation reflects a primary pathway effect or emerges downstream of broader intracellular stress remodeling—an issue that is particularly relevant for flavonoid-rich fractions. One plausible upstream context involves redox remodeling and mitochondrial stress. Flavonoids can shift ROS dynamics in a context-dependent manner, and tumor cells can be especially sensitive to redox perturbation (Schieber and Chandel, 2014; Slika et al., 2022). Consistent with intensified stress at higher exposure, Annexin V/PI quadrant quantification showed that PI-positive cells increased mainly through the Annexin V^+^/PI^+^ population (Q2), whereas Annexin V^−^/PI^+^ cells (Q1) remained minor—a pattern commonly interpreted as accumulation of late-stage PI-positive cells consistent with late apoptosis and/or progression toward secondary necrosis under stronger exposure (Galluzzi et al., 2018). Redox perturbations may also recruit Nrf2-dependent adaptive programs (e.g., HO-1/NQO1 induction), which can reshape survival signaling and apoptosis sensitivity (Kansanen et al., 2013; Hayes and Dinkova-Kostova, 2014). While these upstream processes were not directly measured here, they provide a biologically plausible framework for follow-up testing of how OFE exposure connects stress signaling to PI3K/AKT modulation and caspase activation.

A practical feature of this study is that the cellular assays were performed on an extract prepared under statistically optimized UAE conditions rather than a single empirically chosen setting. The operating-window behavior is consistent with UAE reports that overly harsh temperature or power can compromise phenolic metabolite selectivity or stability (Edmonds and Sancier, 1983; Chaaban et al., 2017), reinforcing the value of defining extraction parameters that avoid diminishing returns and potential degradation (Nadeem et al., 2018).

While the present data provide a pathway-linked *in vitro* phenotype for OFE in a cervical cancer cell model, this work does not yet resolve which individual metabolites drive the effects or whether the upstream redox/Nrf2–mitochondrial axis is causally involved. Future studies will therefore focus on bioactivity-guided fractionation and metabolite identification, targeted validation of ROS/Nrf2/mitochondrial stress, and *in vivo* evaluation to test whether the pathway-associated phenotype persists beyond this initial cell-based context.

## 5 Conclusion

In this study, we optimized an ultrasound-assisted extraction process to obtain a flavonoid-enriched fraction from *Physochlaina physaloides* (L.) G. Don and evaluated its effects in a cervical cancer cell model. OFE reduced HeLa cell viability and increased Annexin V–positive fractions, accompanied by decreased PI3K/AKT phosphorylation and increased caspase-3 cleavage, supporting an apoptosis-associated *in vitro* phenotype. Active-metabolite identification and *in vivo* validation are required before broader conclusions can be drawn; the present findings are limited to cell-based assays of a complex extract mixture but provide preliminary mechanistic insight into apoptosis-associated signaling.

## Data Availability

The raw data supporting the conclusions of this article will be made available by the authors, without undue reservation.

## Supplementary material

SUPPLEMENTARY FIGURE S1 UHPLC–MS/MS base peak chromatogram of OFE acquired in positive-ion mode. Sixteen major peaks were labeled and subjected to preliminary annotation based on Compound Discoverer 3.0 and mzCloud database matching. The corresponding putative compound identities, retention times, molecular formulae, observed *m/z* values, and mzCloud Best Match scores are summarized in Supplementary Table S2. These annotations are tentative and were used to improve the chemical definition of OFE as a chemically complex extract.

SUPPLEMENTARY FIGURE S2 Representative FSC/SSC plots showing the initial gate (P1) used to define the main cell population and exclude debris in HeLa cells treated with OFE at 0, 12.5, 25, and 50 μg/mL prior to Annexin V/PI apoptosis analysis.
