# Supplementary material for: Optimized ultrasound extraction and cytotoxic activity against HeLa cervical cancer cells of total flavonoids of Physochlaina physaloides (L.) G. Don
Source: Front Pharmacol. 2026 Apr 8;17:1800105. doi: 10.3389/fphar.2026.1800105 (PMC13100907; doi:10.3389/fphar.2026.1800105)

***Supplementary Material***

**Supplementary Figures and Tables**

**Supplementary Table S1** MTT viability of non-transformed H8 cervical epithelial cells after OFE treatment at 24, 48, and 72 h

| Time | OD | | | |
| --- | --- | --- | --- | --- |
|  | 12.5 μg/mL | 25 μg/mL | 50 μg/mL | Control |
| 24 h | 0.915  0.884  0.904 | 0.872  0.895  0.868 | 0.822  0.846  0.865 | 0.893  0.905  0.887 |
| 48 h | 0.785  0.802  0.776 | 0.741  0.729  0.733 | 0.715  0.743  0.720 | 0.768  0.749  0.755 |
| 72 h | 0.725  0.708  0.712 | 0.705  0.709  0.716 | 0.723  0.695  0.711 | 0.741  0.726  0.719 |

**Supplementary Methods**

**UHPLC–MS/MS profiling and preliminary constituent annotation of OFE**

For qualitative analysis, 2 g of the flavonoid extract was dissolved in 25 mL of 80% methanol, vortex-mixed thoroughly, and filtered through a 0.22 μm microporous membrane prior to injection.

The chemical profile of the optimized flavonoid extract (OFE) was analyzed using a Thermo Acclaim™ RSLC 120 C18 column (2.1 mm × 150 mm, 2.1 μm). The mobile phase consisted of methanol (A) and 0.1% formic acid in water (B). Gradient elution was performed as follows: 0–2 min, 5–20% A; 2–25 min, 20–95% A; and 25–26 min, 95–5% A. The column temperature was maintained at 30°C, the injection volume was 2 μL, and the flow rate was 0.3 mL/min.

Mass spectrometric detection was carried out on a Q-Exactive Orbitrap high-resolution mass spectrometer (Thermo Fisher Scientific, USA) equipped with a heated electrospray ionization (HESI) source in both positive- and negative-ion modes. Data were acquired in Full MS/data-dependent MS² (Full MS/dd-MS²) mode. The resolution was set at 70,000 for Full MS and 17,500 for dd-MS², with a scan range of *m/z* 80–1,200. The spray voltage was +3.5 kV in positive-ion mode and −3.0 kV in negative-ion mode. The capillary temperature was 320°C, the S-lens RF level was 55, and the vaporizer temperature was 350°C. Sheath gas and auxiliary gas were set at 40 and 10 arbitrary units, respectively. Stepped collision energies of 30, 50, and 70 eV were applied for MS/MS fragmentation.

Raw data were processed using Compound Discoverer 3.0 for database searching against the mzCloud library and were further examined using Thermo Xcalibur software. Compounds with mzCloud Best Match scores >85 were retained as preliminarily annotated constituents.

**Supplementary Table S2** Preliminary annotated constituents of the optimized flavonoid extract (OFE) identified by UHPLC–MS/MS

| No. | t_R_ (min) | Compound | Formula | *m/z* | MzCloud  Best match |
| --- | --- | --- | --- | --- | --- |
| 1 | 3.22 | Epicatechin | C_15_H_14_O_6_ | 290.0790 | 97.1 |
| 2 | 6.19 | Quercetin | C_15_H_10_O_7_ | 302.0427 | 98.6 |
| 3 | 7.10 | Daidzin | C_21_H_20_O_9_ | 416.1107 | 85.9 |
| 4 | 7.68 | Kaempferol | C_15_H_10_O_6_ | 286.0478 | 96.1 |
| 5 | 8.20 | Apigenin-7-O-glucoside | C_21_H_20_O_10_ | 432.1057 | 97.5 |
| 6 | 9.53 | Hyperoside | C_21_H_20_O_12_ | 464.0548 | 94.9 |
| 7 | 10.11 | Naringenin | C_15_H_12_O_5_ | 272.0685 | 86.5 |
| 8 | 10.44 | Naringenin-7-O-glucoside | C_21_H_22_O_10_ | 434.1213 | 96.9 |
| 9 | 11.15 | Kaempferol-3-O-rutinoside | C_27_H_30_O_15_ | 594.5182 | 96.4 |
| 10 | 11.62 | Rutin | C_27_H_30_O_16_ | 610.1534 | 97.7 |
| 11 | 12.09 | Isorhamnetin | C_16_H_12_O_7_ | 316.0538 | 88.2 |
| 12 | 14.36 | Quercitrin | C_21_H_20_O_8_ | 448.1006 | 88.1 |
| 13 | 14.72 | Nobiletin | C_21_H_22_O_8_ | 402.1315 | 85.9 |
| 14 | 18.07 | Umbelliferone | C_9_H_6_O_3_ | 162.0389 | 89.2 |
| 15 | 20.85 | 5,7-Dimethoxycoumarin | C_11_H_10_O_4_ | 206.0652 | 88.0 |
| 16 | 21.91 | Salicylic acid | C_7_H_6_O_3_ | 138.1220 | 91.8 |

**Supplementary Figure S1** UHPLC–MS/MS base peak chromatogram of the optimized flavonoid extract (OFE) with preliminary peak annotation


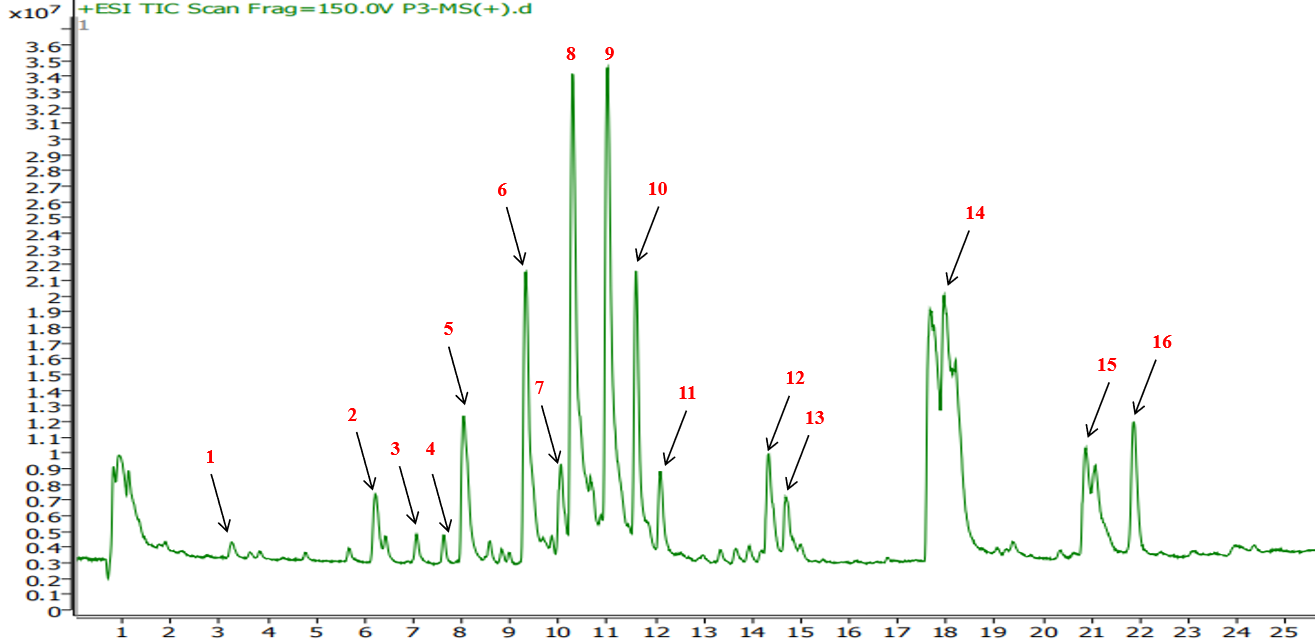


**Supplementary Figure S2** Representative flow cytometry gating strategy for Annexin V/PI apoptosis analysis in HeLa cells after OFE treatment


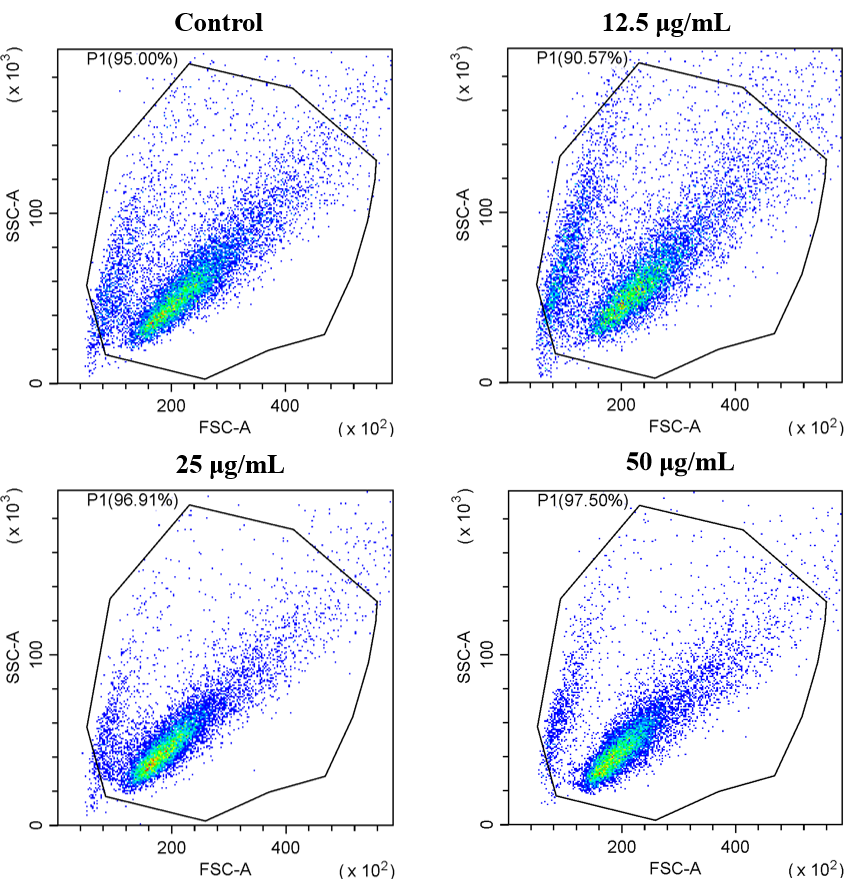

Supplement: Supplementary file 1 [file Supplementaryfile1.docx]
